# Treatment of Cervical Artery Dissection: Antithrombotics, Thrombolysis, and Endovascular Therapy

**DOI:** 10.1155/2017/3072098

**Published:** 2017-05-21

**Authors:** Jing Peng, Zunjing Liu, Chunxia Luo, Lin Chen, Xianhua Hou, Li Xiao, Zhenhua Zhou

**Affiliations:** ^1^Department of Neurology of Southwest Hospital, Third Military Medical University, No. 30 Gaotanyan Road, Chongqing 400038, China; ^2^Department of Neurology, China-Japan Friendship Hospital, 2 Yinghua Dongjie, Hepingli, Beijing 100029, China

## Abstract

Cervical artery dissection (CAD) is an important cause of stroke for young patients, accounting for 5–22% of strokes in patients <45 years of age, which presents not only a great burden to the stroke victims but also a financial burden to the family and society. Because CAD can lead to different clinical lesions, including neuropathy, acute ischemic stroke, and subarachnoid hemorrhage, and is an arterial dissection with a self-healing tendency, the treatment options depend on the clinical manifestations. The main purpose of the treatment is to control CAD-induced neuronal damage and to restore blood flow. The treatment programs include drug treatment and endovascular treatment. However, antithrombotic treatment is crucial. Both antiplatelet drugs and anticoagulant drugs are used to reduce the risk of stroke, but whether one treatment strategy is more effective than the other is unknown. The efficacy and timing of the endovascular treatment of CAD remain controversial.

## 1. Introduction

During cervical artery dissection (CAD), arterial blood enters the blood vessel wall through the damaged carotid intima, which separates the intimal and media layers. This process causes the formation of an intramural hematoma, resulting in stenosis or occlusion. CAD can cause thrombosis and vascular stenosis and is the major cause of stroke among young people. Patients with CAD as the main cause account for 2.5% of all stroke patients and 5–22% of young stroke patients below 45 years of age [1]. Recently, the prevalence of CAD has attracted increasing clinical attention due to the continuous development of imaging technologies. However, the pathogenesis of CAD is still unclear. Despite the fact that treatment options for stroke remain limited [2], disputes on the efficacies of anticoagulation and antiplatelet therapies are increasing, and the efficacy and timing of the endovascular treatment of CAD remain controversial. The aim of this review is to summarize the recent reported results regarding the treatment of CAD.

## 2. Diagnosis of CAD

Mostly CAD patients are young; according to studies in Europe and America, the average age of CAD patients is 44.0–45.8 years [3]. A European hospital-based multicenter study showed that males accounted for 53–57% of CAD patients, whereas a North American population-based study revealed that females accounted for 50–52% of CAD patients [4, 5]. The clinical manifestations of CAD are diverse, with typical features termed the CAD triad (ipsilateral pain in the head, neck, and face, Horner syndrome, and cerebral or retinal ischemic symptoms) [6]. However, less than one-third of patients manifest the triad, and the most common symptoms are headache (70–80%) and cerebral ischemic symptoms (67%) [7]. Notably, approximately 5% of CAD patients are asymptomatic [8]. Ischemic stroke is the most common type of secondary brain vascular disease in CAD patients. Due to the continuous development of medical imaging technologies, the CAD diagnosis largely depends on imaging techniques, such as computed tomography angiogram (CTA), magnetic resonance imaging (MRI), digital subtraction angiography (DSA), and Doppler ultrasound. Of these techniques, DSA has long been considered as the gold standard for CAD diagnosis. In DSA, artery dissection exhibits beaded and thread-like symptoms, irregular fan-shaped stenosis, indirect signs, such as pseudoaneurysm and venous phase contrast agent retention, and direct signs, such as dual chamber symptoms of two-way blood flow [9]. Due to its wide application and noninvasiveness, CTA can provide important information for the diagnosis of CAD. CTA has a false positive rate of 0 for the diagnosis of vascular occlusion and a detection rate of 96% for the diagnosis of vessel wall thickening and irregular changes and is superior to MRI in revealing intimal flaps and pseudoaneurysms [10]. Color Doppler ultrasound can directly show the situation of the arterial wall and detect both direct and indirect signs of CAD. Transcranial Doppler (TCD) is capable of measuring the blood flow velocity and performing arterial emboli monitoring and helps to determine the presence of CAD. In severe carotid artery stenosis or occlusion, the sensitivity of ultrasound can be 100%, whereas, in mild stenosis, the sensitivity drops to 40% [11]. Currently, noninvasive imaging techniques, such as MRI and magnetic resonance angiography (MRA), are playing increasingly important roles in the CAD diagnosis. MRI diffusion-weighted imaging (DWI) can lead to the early detection of CAD-induced cerebral changes. Axial MRI can show the situation on the blood vessel wall or lumen to some extent [12]. MRA experiences less interference from the bone structure and more completely displays vascular structures, especially in the presence of a contrast agent. High-resolution imaging of the vascular wall structure presented by high-resolution MRI (HRMRI) can differentiate the carotid artery and the surrounding tissues, such as the vertebral artery and the surrounding veins, and is more conducive to the identification of a vessel wall hematoma and intravascular thrombus [10].

## 3. Anticoagulant Therapy and Antiplatelet Therapy of CAD

Drug treatment primarily consists of antithrombotic therapy (i.e., anticoagulant and antiplatelet therapies). Anticoagulant therapy includes intravenous heparin therapy coupled with oral application of warfarin, whereas antiplatelet therapy includes a single oral antiplatelet treatment with one antiplatelet aggregation drug or a dual antiplatelet treatment with a combination of two antiplatelet aggregation drugs as the main treatment programs; antiplatelet treatment includes aspirin, dipyridamole, or clopidogrel alone or in combination.

The main rationale for antithrombotic therapy is that transcranial Doppler studies have demonstrated that the frequency of intracranial microembolic events is rather high in CAD patients [10]. After a stroke, antithrombotic therapy needs to be performed immediately to minimize the thrombosis at the dissection site and to reduce CAD-induced neuronal damage. The duration of antithrombotic therapy is generally 3–6 months, and this therapy rarely lasts more than 6 months. There are no clear guidelines regarding the sign for the termination of antithrombotic treatment; generally, vascular imaging characteristics, such as dissection healing or vascular occlusion, are used to develop further therapy programs after antithrombotic treatment [13, 14].

In 2011, the US extracranial carotid and vertebral artery disease management guidelines recommended that symptomatic CAD patients be subjected to oral anticoagulant therapy with warfarin (INR2.0~3.0) for 3 to 6 months after intravenous heparin and then switched to long-term use of aspirin or clopidogrel antiplatelet therapy [15].

At present, there has been a lack of large randomized controlled trials to compare the efficacies of antiplatelet and anticoagulant drugs for the prevention of recurrent stroke in CAD patients. Clinicians should choose treatment options based on personal experience and the patient's specific circumstances. Kennedy et al. conducted a meta-analysis in 2012 in which 40 nonrandomized groups consisting of 1636 cases were analyzed. The results showed that, for the treatment of recurrent stroke risk with antiplatelet and anticoagulant therapies, the outcome was as follows: antiplatelet aggregation drugs 2.6% (13/499) versus anticoagulation drugs 1.8% (20/1137), odds ratio (OR) 1.49, whereas, for the risk of death, the outcome was as follows: antiplatelet aggregation drugs 1.00% (5/499) versus anticoagulation drugs 0.80% (9/1137), OR1.27. However, the differences were not significant, and there was no clear evidence that either anticoagulants or antiplatelet aggregation drugs had an obvious advantage [16]. A meta-analysis conducted by Sarikaya et al. in 2013 obtained similar results, but the authors noted that more emphasis should be placed on the use of antiplatelet aggregation drugs because of their convenience and cost and that they should be recommended as the first-line medication [17]. The Cervical Artery Dissection in Stroke Study (CADISS) was a multicenter prospective randomized controlled study in which the efficacy and safety of antiplatelet and anticoagulant therapies in patients with an acute CAD onset within 7 days were investigated. Rigorous randomized controlled experiments on 250 cases found no significant differences in the efficacies of the anticoagulation and antiplatelet therapies; only 2% of patients had a stroke incidence, which was lower than the incidence reported in other observational studies. However, the study had some limitations; for example, the follow-up time was only three months, the long-term efficacy was not followed up, and the sample size was inadequate. Therefore, the study could not determine the difference between the two treatments [18]. Still, during the study, the researchers conducted an analysis using data from the patient population unsuitable for randomization (CADISS.NR) and a meta-analysis on 40 nonrandomized items, including the CADISS.NR research. The results showed that 499 of the 1636 cases received antiplatelet therapy and 1137 received anticoagulation therapy; the recurrent stroke rates were 2.6% and 1.8% for these two groups and the fatality rates were 1% and 0.8%, respectively. No significant differences were detected between the two treatments [19].

In-depth study of the pathogenesis showed that, in addition to secondary hypoperfusion and an arterial originated embolism caused by thrombosis shedding in the dissection, hemodynamic instability played an important role in the CAD occurrence of intramural hematoma formation [20, 21]. Thus, the use of anticoagulant drugs might cause intramural hematoma expansion and exacerbate abnormal dissection hemodynamics. Moreover, the recurrence rate of CAD-caused ischemic stroke is low, and the persistent risk of bleeding during anticoagulation therapy to some extent offsets the benefits of the anticoagulation therapy. The significance of antiplatelet therapy lies in the prevention of the recurrence of early stroke. Based on clinical experience, the application of antiplatelet therapy has a wider range, including stenosis, occlusion, and pseudoaneurysm. The use of antiplatelet aggregation drugs is also recommended in CAD patients with a poor prognosis or a large number of embolism incidences [13, 22]. Borgess type I and II patients all benefited from dual antiplatelet therapy [23]. Thus, drug treatment programs for patients can be determined by taking into account the following points: (1) anticoagulant therapy should be preferred for CAD patients in the acute stage (within 7 days of the onset) with obvious symptoms (i.e., after intravenous heparin, switch to anticoagulation treatment with oral warfarin (INR2.0~3.0), for 3–6 months); however, antiplatelet therapy needs to be immediately terminated for patients with severe stroke (NIHSS score ≥ 15) complicated with intracranial atherosclerotic disease (ICAD) or local compression symptoms not complicated with stroke/TIA (transient ischemic attack), complicated with diseases with a high risk of bleeding, and complicated with factors such as poor intracranial collateral circulation [13]; (2) based on the extensiveness and the safety of the drug use, antiplatelet therapy should be preferentially adopted for patients with other types of CAD, with dual antiplatelet therapy for 3 months considered appropriate; and (3) quality of life should be improved to control other risk factors.

## 4. Thrombolysis Therapy of CAD

Intravenous thrombolysis is an effective treatment for ischemic stroke [24]. The treatment of acute cerebral infarction using recombinant tissue-type plasminogen activator (rtPA) has proven to be effective in lowering the mortality and morbidity of acute cerebral infarction in multiple large randomized trials [25, 26]. In CAD-induced ischemic stroke, clinicians are often concerned that rtPA thrombolytic therapy may aggravate vascular injury and increase the risk of bleeding. However, only limited cases have been reported to date, and analyses on the efficacy and safety of thrombolysis therapy in patients with CAD-induced ischemic stroke are lacking from randomized controlled studies. In a recent meta-analysis on patients receiving intravenous thrombolysis and arterial therapies in the Safe Implementation of Thrombolysis in Stroke International Stroke Thrombolysis Register (SITS-ISTR) as of March 2010, 180 cases of CAD patients with acute ischemic stroke (with an average NIHSS score of 16) were investigated, of whom 67% received intravenous thrombolysis therapy and 33% received arterial thrombolysis therapy; the outcome was that the overall incidence of intracranial hemorrhage, the overall mortality rate, and the proportion of patients with a good prognosis were 3.1%, 8.1%, and 41%, respectively. Compared with stroke cases caused by other etiologies in the SITS-ISTR, the CAD patients receiving thrombolysis therapies showed no significant differences in terms of safety and prognosis [27]. Thus, we believe that the treatment of CAD-induced acute ischemic stroke using intravenous rtPA within 4.5 h of onset is safe. However, we should strive to develop new therapeutic strategies to lower the mortality and disability rates of CAD patients after thrombolytic therapy [25].

## 5. Endovascular Treatment of CAD

Endovascular treatment has been widely used to treat cardiovascular and cerebrovascular diseases [28]. However, randomized controlled studies on the application of endovascular treatment or surgeries for CAD patients have not been reported to date [29, 30], and the efficacy and safety of endovascular therapy or surgical treatment have not been evaluated in CAD patients. Endovascular treatment has been primarily used in CAD patients with failed antithrombotic treatment with contraindications for anticoagulation and a pseudoaneurysm and when stent implantation is the main vascular interventional procedure. Due to the special pathological physiology of cervical artery dissection, the method of endovascular treatment is cervical artery stenting. Endovascular treatment/surgical treatment for CAD should be limited because CAD patients have a lower risk of recurrent ischemic stroke, there is no significant correlation with CAD-induced vascular stenosis and pseudoaneurysm, and endovascular/surgical treatments are traumatic. With the development of vascular interventional procedures, the application of endovascular treatment in CAD patients may be underestimated; furthermore, it was previously believed that the dissection leads to clinical events mainly through thromboembolism rather than hypoperfusion; thus, antithrombotic therapy has been the preferred treatment for CAD [31]. However, endovascular treatment can also be viewed as the preferred option for the treatment of CAD patients, especially when the patient has both an embolism and obvious hypoperfusion [32]. In this case, endovascular treatment can effectively relieve stenosis, increase blood flow, and improve low perfusion. In a retrospective study, 140 cases of CAD patients received stenting, and angiographic follow-up was conducted for an average of 12.8 months. The results showed that dissection-induced vascular stenosis was significantly improved and that secondary stroke events accounted for only 1.4% of cases. Thus, endovascular therapy could effectively improve CAD-induced vascular stenosis and reduce the incidence of ischemic stroke [33]. Multiple overlapping stents could also effectively reduce the blood flow velocity in pseudoaneurysms and promote thrombosis, thereby shrinking the pseudoaneurysm or causing it to disappear. Previous studies showed that dissection stenosis of CAD patients undergoing stenting therapy could be largely eased, from 71% to complete remission [29]. In terms of the progression of CAD and the structural damage to the vessel wall, patients in the acute stage and Borgess type IB and II patients would significantly benefit from the use of stenting as the preferred treatment [23].

Endovascular treatment also has some specific risks, the most important of which is that the stenting operation in winding vessels is prone to some unforeseen outcomes [34]. Complications of endovascular treatment are numerous and range from mild to severe transient neurological damage and even death, including iatrogenic arterial dissection, peripheral thromboembolism, arterial spasm, stent thrombosis, arterial wall perforation by the guide wire, stent migration, stroke, and endometrial hyperplasia [32]. The timing of endovascular treatment for atherosclerosis is now unclear, especially in the case of dissection. In endovascular treatment, an emboli protection device can effectively reduce the risk of embolism during the procedure [35].

Although endovascular treatment has a higher risk and higher requirements on the operator than drug therapy, CAD patients (especially those in the acute stage) would greatly benefit from the strict control of surgical indications. If drug therapy is ineffective for the patient and the patient can generally withstand surgery and is suggested to have acute cerebral infarction by laboratory examination, stenosis, or occlusion caused by hematoma based on the pathophysiological manifestations, or an expanding dissection lesion, the implementation of endovascular surgery would generate more benefits than risks [33].

## 6. Conclusions and Further Directions

CAD is an important factor that causes stroke in young people. The aim of this review was to summarize the treatment of CAD, and the results are summarized in Table 1. CAD is a disease that has only rarely been diagnosed through autopsy but is now readily diagnosed with the in- depth study of CTA, MRI, and DSA applications. Thus, it is imperative to establish a reasonable and standardized treatment system with few disputes. Because the causes of CAD are not clear, its risk factors need to be discovered. In addition to factors such as high blood pressure and high cholesterol [36], Giossi et al. showed the close correlation between connective tissue abnormalities and the incidence of CAD; interestingly, the association of genetic connective tissue diseases with the occurrence of CAD has not been established [37, 38]. Antiplatelet and anticoagulant therapies currently do not show differences in terms of efficacy, although the cases that have been investigated have been rather limited, and larger scale studies need to be performed. Although the timing of endovascular treatment is still an open question, endovascular treatment will be more widely adopted with further investigations on CAD's secondary injuries. Moreover, the rational use of antihypertensive drugs to control blood pressure in the normal range and to reduce arterial wall pressure is a necessary intervention [1]. Furthermore, the application of statins for the treatment of CAD needs to be addressed; although this approach lacks relevant case studies, Stein et al. comprehensively analyzed 1560 patients with thoracic aortic aneurysms in 2013 and noted that statins played a positive role in the prognosis of aortic aneurysms [39]. Thus, we propose a bold assumption that statins may also have a positive impact on CAD patients, although this hypothesis requires a theoretical basis through more basic experiments with large sample sizes from multicenter randomized controlled trials. Only in this way can we find conclusive evidence for treatment options and eliminate confusion in the treatment of CAD.

## Figures and Tables

**Table 1:** The summary of treatment of CAD.

Therapies	Indications	Advantages	Disadvantages
Antithrombotics	Conventional therapy in the acute and chronic phase	Oral application and good compliance	Void for part of patients

Thrombolysis	For patients within 4.5 h of onset	Reopen an occluded artery quickly	Maybe leading to intramural bleeding

Endovascular therapy	For patients who have definite recurrent cerebral ischemic events despite medical therapy	Higher rates of revascularization	Potential risks, including peripheral thromboembolism, arterial spasm, and stent thrombosis

*Note*. CAD: cervical artery dissection.

## References

[1] Thanvi B., Munshi S. K., Dawson S. L., Robinson T. G. (2005). Carotid and vertebral artery dissection syndromes. *Postgraduate Medical Journal*.

[2] Lioutas V.-A., Alfaro-Martinez F., Bedoya F., Chung C.-C., Pimentel D. A., Novak V. (2015). Intranasal insulin and insulin-like growth factor 1 as neuroprotectants in acute ischemic stroke. *Translational Stroke Research*.

[3] Lee V. H., Brown R. D., Mandrekar J. N., Mokri B. (2006). Incidence and outcome of cervical artery dissection: a population-based study. *Neurology*.

[4] Touzé E., Gauvrit J.-Y., Moulin T., Meder J.-F., Bracard S., Mas J.-L. (2003). Risk of stroke and recurrent dissection after a cervical artery dissection: a multicenter study. *Neurology*.

[5] Arnold M., Kappeler L., Georgiadis D. (2006). Gender differences in spontaneous cervical artery dissection. *Neurology*.

[6] Biller J., Sacco R. L., Albuquerque F. C. (2014). Cervical arterial dissections and association with cervical manipulative therapy: a statement for healthcare professionals from the American Heart Association/American Stroke Association. *Stroke*.

[7] Baumgartner R. W., Arnold M., Baumgartner I. (2001). Carotid dissection with and without ischemic events: local symptoms and cerebral artery findings. *Neurology*.

[8] Flis C. M., Jäger H. R., Sidhu P. S. (2007). Carotid and vertebral artery dissections: clinical aspects, imaging features and endovascular treatment. *European Radiology*.

[9] Naggara O., Louillet F., Touzé E. (2010). Added value of high-resolution MR imaging in the diagnosis of vertebral artery dissection. *American Journal of Neuroradiology*.

[10] Lum C., Chakraborty S., Schlossmacher M. (2009). Vertebral artery dissection with a normal—appearing lumen at multisection CT angiography: the importance of identifying wall hematoma. *American Journal of Neuroradiology*.

[11] Sturzenegger M. (1995). Spontaneous internal carotid artery dissection: early diagnosis and management in 44 patients. *Journal of Neurology*.

[12] Provenzale J. M. (1995). Dissection of the internal carotid and vertebral arteries: imaging features. *American Journal of Roentgenology*.

[13] Debette S., Leys D. (2009). Cervical-artery dissections: predisposing factors, diagnosis, and outcome. *The Lancet Neurology*.

[14] Schievink W. I. (2001). Spontaneous dissection of the carotid and vertebral arteries. *The New England Journal of Medicine*.

[15] Brott T. G., Halperin J. L., Abbara S. (2013). 2011 ASA /ACCF/ AHA/ AANN/ AANS/ ACR/ ASNR/ CNS/ SAIP/ SCAI/ SIR/ SNIS/ SVM/ SVS guideline on the management of patients with extracranial carotid and vertebral artery disease: executive summary. *Catheterization and Cardiovascular Interventions*.

[16] Caplan L. R. (2013). Antiplatelets vs anticoagulation for dissection: CADISS nonrandomized arm and meta-analysis. *Neurology*.

[17] Sarikaya H., da Costa B. R., Baumgartner R. W. (2013). Antiplatelets versus anticoagulants for the treatment of cervical artery dissection: bayesian meta-analysis. *PLoS ONE*.

[18] CADISS Trial Investigators (2015). Antiplatelet treatment compared with anticoagulation treatment for cervical artery dissection (CADISS): a randomised trial. *The Lancet Neurology*.

[19] Kennedy F., Lanfranconi S., Hicks C. (2012). Antiplatelets vs anticoagulation for dissection: CADISS nonrandomized arm and meta-analysis. *Neurology*.

[20] Kim Y.-K., Schulman S. (2009). Cervical artery dissection: pathology, epidemiology and management. *Thrombosis Research*.

[21] Menon R., Kerry S., Norris J. W., Markus H. S. (2008). Treatment of cervical artery dissection: a systematic review and meta-analysis. *Journal of Neurology, Neurosurgery and Psychiatry*.

[22] Engelter S. T., Brandt T., Debette S. (2007). Antiplatelets versus anticoagulation in cervical artery dissection. *Stroke*.

[23] Al-Ali F., Perry B. C. (2013). Spontaneous cervical artery dissection: the Borgess classification. *Frontiers in Neurology*.

[24] Zhong G., Yan S., Zhang S., Chen Q., Lai Y., Lou M. (2016). Association between leukoaraiosis and poor outcome is not due to reperfusion inefficiency after intravenous thrombolysis. *Translational Stroke Research*.

[25] Weintraub M. I. (2006). Thrombolysis (tissue plasminogen activator) in stroke: a medicolegal quagmire. *Stroke*.

[26] Morihara R., Kono S., Sato K. (2016). Thrombolysis with low-dose tissue plasminogen activator 3-4.5 h after acute ischemic stroke in five hospital groups in Japan. *Translational Stroke Research*.

[27] Zinkstok S. M., Vergouwen M. D. I., Engelter S. T. (2011). Safety and functional outcome of thrombolysis in dissection-related ischemic stroke: a meta-analysis of individual patient data. *Stroke*.

[28] Lapchak P. A. (2015). Critical early thrombolytic and endovascular reperfusion therapy for acute ischemic stroke victims: a call for adjunct neuroprotection. *Translational Stroke Research*.

[29] Kadkhodayan Y., Jeck D. T., Moran C. J., Derdeyn C. P., Cross D. T. (2005). Angioplasty and stenting in carotid dissection with or without associated pseudoaneurysm. *American Journal of Neuroradiology*.

[30] Joo J. Y., Ahn J. Y., Chung Y. S. (2005). Treatment of intra- and extracranial arterial dissections using stents and embolization. *CardioVascular and Interventional Radiology*.

[31] Ali M. S., Amenta P. S., Starke R. M. (2012). Intracranial vertebral artery dissections: evolving perspectives. *Interventional Neuroradiology*.

[32] Vazquez Rodriguez C., Lemaire V., Renard F., De Keuleneer R. (2005). Primary stenting for the acute treatment of carotid artery dissection. *European Journal of Vascular and Endovascular Surgery*.

[33] Pham M. H., Rahme R. J., Arnaout O. (2011). Endovascular stenting of extracranial carotid and vertebral artery dissections: a systematic review of the literature. *Neurosurgery*.

[34] Albuquerque F. C., Han P. P., Spetzler R. F., Zabramski J. M., McDougall C. G. (2002). Carotid dissection: technical factors affecting endovascular therapy. *Canadian Journal of Neurological Sciences*.

[35] Berkhemer O. A., Fransen P. S. S., Beumer D. (2015). A randomized trial of intraarterial treatment for acute ischemic stroke. *The New England Journal of Medicine*.

[36] Pezzini A., Caso V., Zanferrari C. (2006). Arterial hypertension as risk factor for spontaneous cervical artery dissection. A case-control study. *Journal of Neurology, Neurosurgery and Psychiatry*.

[37] Giossi A., Ritelli M., Costa P. (2014). Connective tissue anomalies in patients with spontaneous cervical artery dissection. *Neurology*.

[38] Debette S., Goeggel Simonetti B., Schilling S. (2014). Familial occurrence and heritable connective tissue disorders in cervical artery dissection. *Neurology*.

[39] Stein L. H., Berger J., Tranquilli M., Elefteraides J. A. (2013). Effect of statin drugs on thoracic aortic aneurysms. *American Journal of Cardiology*.

